# Impact of climatic conditions projected at the World Cup in Qatar 2022 on repeated maximal efforts in soccer players

**DOI:** 10.7717/peerj.12658

**Published:** 2021-12-22

**Authors:** Wiktor Chodor, Paweł Chmura, Jan Chmura, Marcin Andrzejewski, Ewa Jówko, Tomasz Buraczewski, Adrian Drożdżowski, Andrzej Rokita, Marek Konefał

**Affiliations:** 1 Department of Team Games, Wrocław University of Health and Sport Sciences, Wrocław, Dolny Śla̧sk, Poland; 2 Department of Human Motor Skills, Wrocław University of Health and Sport Sciences, Wrocław, Dolny Śląsk, Poland; 3 Department of Recreaction, Poznań University of Physical Education, Poznań, Wielkopolskie, Poland; 4 Department of Natural Sciences, Józef Piłsudski University of Physical Education in Warsaw, Faculty of Physical Education and Health in Biała Podlaska, Podlaskie, Poland; 5 Department of Sport Science, Józef Piłsudski University of Physical Education in Warsaw, Faculty of Physical Education and Health in Biała Podlaska, Podlaskie, Poland

**Keywords:** Soccer, Repeated sprint ability (RSA), Climatic conditions, Heat stress, Hyperthermia, Maximal power, Mechanical parameters, Biochemical parameters, Blood, RPE

## Abstract

This study aimed to investigate the relationship between the climatic conditions predicted for the 2022 FIFA World Cup in Qatar and the capacity for repeated maximum effort (RSA), of soccer players. Twenty-four semi-professional soccer players participated in the study. The exercise test consisted of ten 6-second maximal efforts on a cycloergometer. A 90-second passive rest interval was used. Mechanical parameters were recorded in each repetition, and biochemical parameters at rest and even repetitions. The test was performed in a Weiss Technik WK-26 climate chamber under two different conditions: (1) thermoneutral (TNC - 20.5 °C; 58.7% humidity); (2) predicted for the 2022 World Cup in Qatar (QSC - 28.5 ± 1.92 °C; 58.7 ± 8.64% humidity). Significantly higher mean maximum power values were recorded in the second repetition under QSC conditions (1731,8 ± 214,4 W) (*p* = 0.025). A significantly longer time to reach maximum power was also recorded under TNC conditions compared to QSC conditions in repetition 2 (1,32 ± 0,33 s), (1,05 ± 0,29 s) (*p* = 0.016) and 6 (1,41 ± 0,48 s), (1,17 ± 0,25) (*p* = 0.036). There was a significantly higher rate of power loss, between repetition 2 (*p* = 0.023) and 4 (*p* = 0.043) under QSC conditions, compared to TNC. Considering the biochemical parameters, a significantly higher pO_2_ concentration was registered under QSC conditions in the 10th repetition (*p* = 0.006). The ambient temperature during exercise should be taken into account to determine the anaerobic exercise capacity of the athletes. At higher temperatures, there is a greater capacity for maximal effort, in terms of maximal power achieved, but with a greater decrease in performance.

## Introduction

Nowadays, soccer is more intensified than in the last decade (Barnes et al., 2014). Professional soccer players need to cover longer distances at high and maximum intensity and be more technically advanced (Konefał et al., 2019b). In modern day soccer, it is important to understand sport-specific physical activity demands for performance development and injury prevention of the game. Nowadays, soccer players run ∼11 km per game with 30–40 short sprints every 90 s, which depends on the position (Andrzejewski et al., 2016). During a soccer game, short-lasting movements are performed at both maximum (sprinting, jumping, sliding) and high intensity (counter attacking) (Racinais, Cocking & Périard, 2017). Such movements are characterized by an anaerobic energy metabolic process (Barnes et al., 2014; Andrzejewski, Chmura & Pluta, 2015). This clearly indicates the need to introduce training units in the training process taking into account the formation of the ability for short repeated maximal efforts (RSA).

Besides, RSA generating high power over a short distance and high running speed are among the fundamental abilities of a soccer player (Amonette et al., 2014; Bangsbo, Iaia & Krustrup, 2007; McIntyre, 2005). Chmura et al., (2017a) observed that soccer players at the 2014 World Cup in Brazil, depending on the round of the tournament, reached an average of 27.66 ± 2.32 km/h in the first match of the group stage, up to 28.50 ± 1.82 km/h in the final and third place match. Furthermore, straight sprinting enables a player to escape from his opponent and/or to reach a free zone to shoot for the goal or to give a decisive pass. Goals were mostly scored by forwards after a straight sprint without the ball (Faude, Koch & Meyer, 2012). Additionally, analyses from recent years indicate that the covered sprint distance and number of sprints performed are of major significance in modern soccer (Andrzejewski et al., 2017; Konefał et al., 2019a). During these short duration maximum power efforts, anaerobic energy reactions are the main source of ATP resynthesis (Górski, 2006). Therefore, it is important to monitor the mechanical and biochemical parameters associated with high and maximal intensity exercises to optimally adjust the training program (Andrade et al., 2016).

Amid the effects of global warming, heat-related health risks are increasing especially in sporting events. Global warming is believed to be an ongoing phenomenon, and thermal environment management, particularly heat stress, is growing in importance. Caution is therefore needed to help guard against these health risks during World Cup in Qatar 2022 (Kakamu et al., 2017). High ambient temperatures can be unfavourable for players during major tournaments. This could be seen at the 2014 World Cup in Brazil, Brazil’s climate was a concern for many soccer teams of different nationalities, especially European ones. At that time, the introduction of additional cooling and hydration breaks was considered (Kakamu et al., 2017; Lucena et al., 2017). Also the last IO in Tokyo in 2021 showed that extra breaks are a good solution for athletes to maintain a good sport level. Hence, the investigation of maximal efforts at high temperatures is justified.

The global climate is systematically warming and soccer players are increasingly forced to play in high temperatures (Nassis et al., 2015). According to many authors, the ability to perform high-intensity exercise at high temperatures is significantly reduced and can have a negative impact on sports performance and overall player performance (Grantham et al., 2010; Mohr et al., 2010; Mohr et al., 2012; Ozgünen et al., 2010). A study by Nassis et al. (2015) found that athletes playing in temperatures above 28 °C experienced “heatstress” and reduced high intensity activity and sprinting numbers. At the same time, they maintained their maximum running speed. Similar results were observed by Chmura et al. (2017b) found that during the 2014 World Cup in Brazil, the conditions most unfavourable to physical activity on the part of the players occurred at temperatures above 28 °C and relative humidity above 60%. Removal of excess heat from the body at high ambient temperatures occurs due to dilation of skin blood vessels and increased dermal blood flow, in effect this accelerates heat transfer from the body’s interior to its surface. The evaporation of sweat from the skin surface involves the loss of large amounts of heat. Evaporating 1 l of sweat removes 580 kcal of heat from the body. It is also important to remember that with sweat, apart from water, electrolytes are lost and this in turn affects muscle contractility (Górski, 2006).

Considering the rate of reaching maximal power, it may be higher in a higher temperature environment because a warmed muscle in the optimal temperature range has better contractile capacity (Mohr et al., 2010; Racinais et al., 2005). Girard, Bishop & Racinais (2013) reported a decrease in maximal power (Pmax) from 1 to 10 repetitions, in 6 - second repeated sprints, in both thermoneutral (TNC) and high temperature (HOT) conditions, while the mean Pmax, was higher in HOT conditions, with a similar rate of power decrease in both conditions. In contrast, Drust et al. (2005) reported greater power decreases during repeated maximal efforts in HOT (40 °C) compared to TNC (20 °C). Regarding match exercise, data from the study by Mohr et al. (2012) and Mohr, Krustrup & Bangsbo (2003) showed that well-trained soccer players have a moderate decrease in the total distance covered (7%) and a marked decrease (26%) in the amount of high-intensity running when the game is played in HOT compared to TNC. In contrast, the peak sprint speed was higher in HOT. Furthermore, in monitoring the training process, one of the most popular indicators to control physical load is the Rating Perceived Exertion (RPE) scale, which is strongly correlated with heart rate and blood lactate levels (Laursen & Buchheit, 2019; Billaut & Smith, 2010). Périard et al. (2020) studied 6 s repeated maximal efforts under TNC (20 °C) and HOT (40 °C) conditions and reported no significant differences in RPE. Analysing mechanical parameters is an important element in soccer so that coaches and training staffs have a better knowledge of how players generate high power, which translates into acceleration and deceleration (Dolci et al., 2020).

High-intensity exercise also causes large changes in metabolite and ion concentrations within skeletal muscle (Robergs, Ghiasv & Parker, 2004). During maximal exercise, there is a sharp increase in muscle lactate concentration, indicating energy production from anaerobic glycolysis. Consequently, this results in a decrease in pH levels and disturbances in acid–base balance. Mohr et al. (2012) comparing TNC (21 °C) and HOT (43 °C) matching conditions, reported no significant differences in blood lactate concentration. Similar observations were reported by Yamaguchi et al. (2020); they recorded no significant differences between repeated maximal efforts at TNC (20 °C) and HOT (35 °C) in blood biochemical parameters such as lactate concentration, pH, HCO_3_, pO_2_, despite changing values over time. To the best of the authors’ knowledge, there are very few new publications, *e.g.*, pO_2_ and pCO2, in terms of the topic undertaken. Coaches and training staff are constantly looking for reserves in the modern training process and in particular in RSA. From a practical point of view, knowledge of the influence of different climatic conditions on changes in blood biochemical parameters can be significant in the training process, preparing for competition in a specific climate.

In this investigation, we attempted to investigate the relationship between RSA and simulated climatic conditions with different ambient temperatures. This is the first study to determine the mechanical and biochemical parameters that will be achieved under the climatic conditions anticipated at the 2022 World Championships in Qatar. A better understanding of the relationship of the effect of high ambient temperature on the ability to perform repeated maximal efforts will contribute to a more optimal and beneficial preparation of athletes for competitions held in habitually high temperature locations. High ambient temperatures can be unfavorable for players during major tournaments. Also, the recent Tokyo 2021 IO has shown that high ambient temperatures are very troublesome for athletes, and for this reason extra cooling breaks were introduced to maintain a high level of athletic performance. Therefore, it is reasonable to conduct an experiment of repeated maximal efforts at high temperatures. Hence, the aim of this study was to investigate the relationship between the climatic conditions predicted for the 2022 FIFA World Cup in Qatar and the repetitive maximum effort (RSA) capacity, of soccer players. It was hypothesised that under conditions with higher temperatures, soccer players would achieve higher maximum power and a faster decline in maximum power would be observed. Furthermore, higher ratings of perceived exertion would be recorded, with biochemical parameters indicating greater fatigue changes.

## Materials & Methods

### The experimental approach to the problem

The study protocol consisted of 10, 6-second repeated efforts at maximum intensity, with a 90-second rest interval (Billaut & Smith, 2010; Kappenstein et al., 2015; Mendez-Villanueva, Hamer & Bishop, 2008; Racinais et al., 2007). The athletes performed the same protocol under two different simulated conditions in a climate chamber. The study compared mechanical and biochemical parameters under thermoneutral conditions and under conditions predicted for the 2022 World Cup in Qatar. To predict conditions at the World Cup, the authors calculated climate data occurring over the past 10 years (in the period from 2008 to 2018) on 21 November in Doha, Qatar, from the climate service: https://en.tutiempo.net/. An attempt was made to test selected parameters for RSA and the reaction of soccer players in relation to various conditions that can be accurately reproduced in a climatic chamber.

### Participants

The study involved semi-professional 24 soccer players from a sports club playing in the Polish 4th League. Mean body height among the players was 179.77 ± 6.16 cm, mean body mass 76.02 ± 5.64 kg, and mean age 21.31 ± 3.63 years. The players had 9.0 ± 1.3 years of training experience and trained five times a week. The study was conducted in November 2018 during the starting period of the 2017/2018 season. Inclusion criteria: We included complete data of players that showed all the results in each of the tests used in the present study, aged over 18 years, without cognitive alterations, without recent surgeries and without injuries. Exclusion criteria: players who were unable to complete the test or who had limitations during the study, mainly for health reasons, duly certified by doctors. All playing position from the field were analyzed. The goalkeepers were excluded from the research of the selected team. Also, the participants were instructed not to intake alcohol or drugs for at least 24 h before the games and measures, and were maintaining normal diets.

All participants were briefed with a detailed explanation of the proposed study and its requirements. They were informed of potential risks and provided with written consent forms. Participants were free to withdraw at any time, without any repercussions. The study was conducted according to the guidelines of the Declaration of Helsinki, and approved by the Ethical Review Board of Wrocław University of Health and Sport Sciences (937/17, 27.06.2017). The study conformed to the requirements stipulated by the Declaration of Helsinki, and all health and safety procedures were complied with.

### Procedures

The exercise protocol consisted of 10, 6-second maximal efforts on a MONARK LT2 cycloergometer (Vansbro, Sweden). A 90-second passive rest interval was used between efforts. The subjects’ task was to achieve maximum anaerobic power on the cycloergometer in each repetition. Toe clips and heel straps at the pedals were used for foot fixation. Strong verbal motivation was provided during each exercise. All sprints were performed from the same starting pedal position with the right crank arm positioned 45° forward to the vertical axis. During 90 s of rest after each even sprint, the subjects sat on the cycloergometer, during which capillary blood was drawn.

Before the start of each maximal exercise series, the athlete, after entering the chamber, spent the first 10 min adapting at rest to the conditions in the chamber (Mohr, Krustrup & Bangsbo, 2003), followed by a 5-minute warm-up on the cycloergometer, during which the athlete achieved a heart rate of 150 bpm (Mendez-Villanueva et al., 2012; Edge et al., 2006). 5 min after completion of the warm-up, the actual measurement of the research protocol began. The tests took place from 9 am to 2 pm from Monday to Friday. The study series were separated by 7 days of rest.

The test was performed in a Weiss Technik WK-26 climatic chamber under two different climatic conditions: (1) under thermo-neutral conditions (TNC - ambient temperature 20.5 °C and relative humidity 58.7%); (Ozgünen et al., 2010; Drust et al., 2005), (2) under climatic conditions corresponding to the average of the maximum values of ambient temperature (QSC - 28.5 ± 1.92 °C and relative humidity 58.7 ± 8.64%) occurring in the last 10 years on November 21 in Doha, Qatar, from climate service: https://en.tutiempo.net/.

### Mechanical parameters

The ergometer was interfaced with an IBM-compatible computer system to allow for the collection of data for the calculation of power generated on each flywheel revolution and work performed during each individual sprint repetition (Lab-VIEW, National Instruments Corp., Austin, TX, USA). The power output of the air-braked cycle ergometer is proportional to the cube of the flywheel velocity (Mendez-Villanueva et al., 2012). Instantaneous work was recorded every 0.2-s during each sprint. Work done was then totalled foreach a sprint to determine work (TW) and expressed relative to time to determine the average power (W). Peak power output was calculated from the highest power output recorded in 0.2-s measuring epoch. Before testing, the ergometer was dynamically calibrated on a mechanical rig across range of power outputs (100–2000 W). Data were collected as previously described by Mendez-Villanueva et al. (2012) and in another studies (Billaut & Smith, 2010; Maxwell et al., 1998). The following mechanical parameters were recorded in the study: maximum power (Pmax), time to reach maximum power (tmax), total work (TW), power drop index (Fi). RPE was also recorded. Since the biochemical parameters were tested in even repetitions, as a result of the adopted protocol, only the repetitions in which all measurements overlapped were accepted for analysis.

### Biochemical parameters

The biological material was collected by qualified professional laboratory staff. All the safety rules were strictly adhered to during the time of blood collection. Capillary blood samples, which were drawn from the fingertips of the nondominant hand using a disposable Medlance^®^ Red lancet spike (HTL-Zone, Germany) with a 1.5 mm blade and 2.0 mm penetration depth. 65 µl of blood was collected in the heparinized capillary immediately before warming up and after repeated repetitions of maximal effort. Then the blood sample was transported immediately after it was taken to the Roche Diagnostics Cobas b 123 gas analyzer. The parameters of the acid–base balance were determined, such as: pH, pCO2 - carbon dioxide partial pressure, pO2 - oxygen partial pressure, HCO3 - bicarbonate concentration, La- - lactate concentration. Biochemical parameters were recorded at rest, after 2, 4, 6, 8, and 10 repetitions.

### Statistical analysis

The conformity of the distribution of all examined parameters was checked against normal distribution. The normality of distribution was assessed with the Shapiro–Wilk test (*p* < 0.05). Arithmetic means and standard deviations were calculated for all parameters. An analysis of variance with replication was performed (repeated measures ANOVA), with taking into account factor the climatic conditions: thermoneutral - TNC and projected in Qatar-QSC. Fisher LSD (Least Significant Difference) post-hoc tests were performed to assess differences between means. Moreover, Cohen d effect size was calculated (Maxwell et al., 1998). Effect size were classified as trivial (*d* < 0.2), small (0.2 ≤*d* < 0.5), medium (0.5 ≤*d* < 0.8) and large (*d* > 0.8). The level of statistical significance was set at *p* < 0.05. All statistical analyses were carried out with the use of the STATISTICA 13.3 software package (Dell Inc., Tulsa, OK, USA).

## Results

### Comparison between climatic conditions

Analysis of the protocol of repeated maximal efforts under two different climatic conditions showed that, for the mechanical parameters, significantly higher mean values of maximal power were registered in the second repetition under the conditions predicted in Qatar in 2022 (*p* = 0.025; *d* = 0.75) (Table 1). Furthermore, a significantly longer time was registered to obtain the maximal power under thermoneutral conditions compared to Qatar conditions in repetition 2 (*p* = 0.016) and 6 (*p* = 0.036; *d* = 0.82). In addition, a significantly higher rate of power loss between repetition 2 (*p* = 0.023; *d* = 0.97) and 4 (*p* = 0.043; *d* = 0.65) under the conditions predicted in Qatar was demonstrated. It was also observed that total work (TW), was higher in each repetition in the QSC, although there were no statistically significant differences between conditions.

**Table 1:** Mechanical parameters and RPE under simulated climatic conditions (mean ± SD).

**Parameters**	**Conditions**	**Rep 2 (2)**	**Rep 4 (4)**	**Rep 6 (6)**	**Rep 8 (8)**	**Rep 10 (10)**	**F (sig)**	**SSD (*p* ≤ 0.05)**
P_max_ [W]	TNC	1577,1* **±196,7**	1597,55 ±190,66	1570,05 ±246,71	1623,38 ±197,28	1634,17 ±217,22	2,573 (0,117)	2QSC> 2 TNC
	QSC	1731,8* **±214,4**	1706,69 ±219,85	1694,66 ±247,89	1673,28 ±214,20	1679,45 ±177,34		
TW [J]	TNC	7020,11 ±632,50	7002,19 ±682,72	6854,90 ±829,77	6887,83 ±781,04	6929,35 ±794,39	0,380 (0,541)	
	QSC	7292,51 ±834,35	7131,87 ±900,14	7104,77 ±1023,8	6985,61 ±970,58	6977,51 ±975,04		
t_max_ [s]	TNC	1,32* ±0,33	1,19 ±0,39	**1,41**$**±0,48**	1,21 ±0,30	1,29 ±0,42	**5,491 (0,024)**	2 TNC> 2 QSC 6 TNC> 6QSC
	QSC	1,05* **±0,29**	1,19 ±0,32	**1,17**$ **±0,25**	1,07 ±0,31	1,09 ±0,31		
Fi [%]	TNC	42,07* ±9,35	**44,54#** **±12,43**	43,53 ±12,63	48,24 ±12,49	46,94 ±14,43	**5,853 (0,020**)		2 QSC> 2 TNC 4 QSC> 4 TNC
	QSC	51,12* ±9,48	**52,60#** **±14,77**	49,72 ±10,77	52,91 ±11,99	52,11 ±16,55			
RPE [scale]	TNC	2,59 ±1,01	3,59 ±1,40	4,77 ±1,31	5,32 ±1,55	6,09 ±1,57	0,380 (0,541)		
	QSC	2,58 ±1,26	3,79 ±1,25	4,74 ±1,32	5,74 ±1,28	6,74 ±1,59		

**Notes.**

* Statistical significance difference in 2nd repetition.

# Statistical significance difference in 4rd repetition.

$ Statistical significance difference in 6th repetition.

Conditions TNC Termoneutral QSC projected in Qatar SSD Statistical significance difference Rep. Repetition P_max_ maximal power output TW total work t_*max*_ time to reach maximum power Fi power drop index RPE Rating Perceived Exertion

Considering the analysed gasometric indices, a significantly higher pO2 concentration was registered under the conditions predicted in Qatar in the 10th repetition (*p* = 0.006; *d* = 0.81)—Table 2. On the other hand, there were no significant differences between the climatic conditions in the other parameters.

**Table 2:** Blood gas parameters under simulated climatic conditions (mean ± SD).

**Par** **ameters**	**Conditions**	**Rest**	**Rep 2 (2)**	**Rep 4 (4)**	**Rep 6 (6)**	**Rep 8 (8)**	**Rep 10 (10)**	**F (sig)**	**SSD** **(*p* ≤ 0.05)**
pH	TNC	7,40 ±0,02	7,33 ±0,02	7,29 ±0,03	7,28 ±0,04	7,28 ±0,04	7,27 ±0,04	0,00 (0,891)	
	QSC	7,41 ±0,01	7,33 ±0,03	7,30 ±0,03	7,29 ±0,04	7,29 ±0,04	7,27 ±0,05		
pCO_2_ [mmHg]	TNC	43,11 ±2,73	42,01 ±2,92	40,76 ±2,73	38 ±2,46	36,44 ±2,69	34,5 ±2,80	4,065 (0,054)	
	QSC	41,62 ±2,51	40,24 ±3,57	38,36 ±3,57	35,96 ±3,46	33,61 ±3,46	33,53 ±2,32		
pO_2_ [mmHg]	TNC	71,33 ±6,26	72,92 ±4,12	75,77 ±5,25	78,85 ±6,21	80,97 ±5,07	**90,98**¥**±5,83**	**5,630 (0,023)**	10 QSC> 10TNC
	QSC	74,84 ±5,62	75,04 ±6,10	78,36 ±5,42	79,67 ±5,29	83,78 ±5,09	**95,71**¥**±4,27**		
HCO_3_ [mmol/l]	TNC	26,44 ±1,56	21,23 ±2,00	19,34 ±1,72	18,19 ±1,91	17,46 ±1,82	16,54 ±2,21	1,286 (0,268)	
	QSC	26,01 ±1,37	20,93 ±2,61	18,73 ±1,77	17,6 ±2,36	16,71 ±2,49	15,89 ±2,30		
La- [mmol/l]	TNC	1,92 ±0,40	7,37 ±1,89	9,82 ±2,10	11,43 ±2,62	12,28 ±2,97	13,31 ±2,74	0,008 (0,928)	
	QSC	1,82 ±0,58	7,37 ±2,37	10,1 ±2,36	11,44 ±2,41	12,27 ±2,07	12,73 ±2,88		

**Notes.**

Legend ¥ statistical significance difference in 10th repetition

Conditions TNC Termoneutral QSC projected in Qatar SSD Statistical significance difference Rep. Repetition pH pH scale pCO_2_ carbon dioxide partial pressure pO_2_ oxygen partial pressure HCO_3_ bicarbonate concentration La- lactate concentration

## Discussion

The aim of the current experiment was to investigate the relationship between the climatic conditions predicted for the 2022 World Cup in Qatar and the capacity for repeated maximum effort (RSA), of soccer players. The current study provides a better understanding of the effects of different climatic conditions on RSA, which will be particularly relevant in preparation for the 2022 World Cup in Qatar. Our main findings of this study were higher maximal power values and shorter maximal power onset times occurred under QSC climatic conditions, with a higher rate of power loss than under TNC conditions. Increased whole-body temperature enhances explosive skeletal muscle performance (*e.g.*, sprint and jump) by improving metabolic and contractile function, nerve conduction, and conformational changes associated with muscle contraction. This was observed in our study in achieving maximal power. In contrast, it is associated with increased fatigue development during prolonged maximal voluntary isometric contractions and impairs aerobic capacity (Racinais, Wilson & Périard, 2017). Heating up carries health risks. Indeed, the development of hyperthermia increases the risk of exercise-induced heat illness. The development of whole-body hyperthermia impairs neuromuscular function with alterations occurring at both the central and peripheral level. From a central perspective, elevated heat stress can lead to a reduction in voluntary muscle activation and the loss of force production capacity. We could observe this phenomenon in the fatigue index (Linnane et al., 2004). Considering mechanical parameters, similar results were recorded by Girard, Bishop & Racinais (2013), the average Pmax was 3.1% higher in HOT than in TNC. Moreover, Yamaguchi et al. (2020), reported 3% higher Pmax in HOT than in TNC. Linnane et al. (Racinais & Oksa, 2010) investigating longer 30 s maximal efforts also reported higher Pmax in HOT than in TNC. All these observations are consistent with ours. The tested soccer players performing the research protocol in QSC achieved higher values in these parameters in each repetition. The afore mentioned authors did not record tmax, and our study shows that it is definitely shorter in QSC than in TNC. Interestingly, in our study the highest Pmax in TNC was reached in the last 10th repetition and in QSC in the 2nd repetition. This may be indicative of the different temperatures of the working muscles. This is contrary to the results of Girard, Bishop & Racinais (2013) and Yamaguchi et al. (2020), as they recorded the highest Pmax values in both conditions, at the first repetition. On the other hand, our observations of Pmax and tmax are in line with previous studies describing a thermo-local increase in muscle temperature as a factor that improves muscle contractility, leading to higher energy production, thus better performance during RSA (Racinais et al., 2005; Racinais, Hue & Blonc, 2004; Krustrup et al., 2006). In our study, analysing mechanical parameters, we also registered a significantly higher Fi in QSC than in TNC. In contrast, Girard, Bishop & Racinais (2013) registered a greater Fi in TNC -19.7% than in HOT -16.5%, between 1 and 10 repetitions, which is the opposite result to ours. In contrast, Drust et al. (2005) recorded greater power decreases in HOT than in TNC. In the available literature, to date, in studies of 6-second efforts, RPE after each single exercise under different climatic conditions has not been frequently recorded (Billaut & Smith, 2010; Kappenstein et al., 2015; Mendez-Villanueva, Hamer & Bishop, 2008; Racinais et al., 2007). Périard et al. (2020), reported no significant differences in RPE between HOT and TNC. In our study, there were also no significant differences in this parameter between QSC and TNC.

In the study by Mohr et al. (2012) showed that the total distance sprinted was the same in HOT - 43 °C and TNC - 21 °C, while the peak and average velocity was higher in HOT than TNC conditions. Which is confirmed by our study as we recorded significantly higher Pmax in QSC. Mohr et al. (2010) revealed an almost 3% decrease in the performance of repeated 30 m sprints at an ambient temperature of 31.6 °C, which is similar to the 2–4% decrease observed during the matches analysed by Krustrup et al. (Mohr et al., 2006). Mohr et al. (2010) found that even in well-trained elite athletes, the ability to perform repeated high-intensity sprints and runs in the last 15 min of a match decreased significantly in HOT, induced by increasing fatigue. Hence, we can predict how soccer players will respond to high temperatures during the 2022 World Cup in Qatar. Therefore, further studies should look at the analysis of parameter changes over time.

When analysing the biochemical parameters, we found no significant differences between the climatic conditions, which may suggest that fatigue levels were very similar in both conditions. The only biochemical parameter in which there were significant changes was oxygen partial pressure, in the 10th repetition. To the best of the authors’ knowledge, there are very few new publication, *e.g.*, pO_2_ and pCO_2_, in terms of the topic undertaken. Yamaguchi et al. (2020), recorded no significant differences in RSA between TNC (20 °C) and HOT (35 °C) in blood biochemical parameters such as lactate concentration, pH, HCO_3_, pO_2_. Furthermore, Mohr et al. (2012) comparing TNC (21 °C) and HOT (43 °C) matching conditions, recorded no significant differences in blood lactate concentration. In addition, in our study we did not register significant differences between QSC and TNC in this biochemical parameter. It is worth noting in particular that in the study by Yamaguchi et al. (2020), there was a 15 °C difference between HOT and TNC and in Mohr’s (Mohr et al., 2012) as much as 22 °C difference and yet there was no difference between climatic conditions in biochemical parameters. The implication is that the temperature difference does not modify biochemical responses for short 6 s repeated maximal efforts, perhaps it is too short effort for significant differences to occur between climatic conditions. Therefore, further studies analysing the body’s response to RSA under different climatic conditions are needed.

Kakamu et al. (2017) noted that acclimatization to heat can be critical in preventing heat illness, as it increases tolerance, adjusts the body’s sweating mechanism (such as the threshold body temperature for sweating), causes excessive sweating, and decreases the amount of sodium lost through sweat. Recently, heat acclimatization before competing in the heat has become an important measure for athletes, and heat acclimation has also become part of the training regimen for sports competition (Kakamu et al., 2017). Given that the weather cannot be changed (external and non-modifiable factor), heat preparedness efforts should focus on modifiable and internal personal factors (*e.g.*, acclimatization, hydration, nutrition, pre-cooling), as well as allowing breaks when needed and lowering temperatures in warm-up areas and lowering temperatures in competition areas (Vanos et al., 2020).

A limitation of this study was that it was conducted in a climate chamber, a nonspecific environment for soccer players, and on a cycloergometer, which is not a characteristic exercise for soccer, and without a game-related stressor. Hence, the conclusions of this study should be used with caution. In further research, it would be advisable to carry out an experiment on different age groups and different sport levels, under natural conditions typical of soccer - on a grass pitch and a running effort, with different lengths of rest breaks. It would be possible to conduct such a study under a specialised balloon, simulating specific climatic conditions within it. Other biochemical indicators, both peripheral and central, could be used to better characterise the resulting fatigue. The strength of this study is primarily, well-defined conditions in a climate chamber were created to address the predicted climatic conditions that will prevail during the 2022 World Cup in Qatar. This is the first study that the capabilities of soccer players in terms of mechanical parameters and biochemical responses of the body were analyzed, in terms of the frequency of sprints performed by players in the game.

## Conclusions

The results of this study suggest that the ambient temperature in which training sessions are conducted and matches played should be taken into account to predict the players’ exercise capacity and adopt an appropriate playing strategy. Coaches and training staffs should be mindful of the players’ greater ability in terms of power output at higher temperatures, while being mindful of the more rapid onset of fatigue and performance decline. On the other hand, the temperature difference between the climatic conditions, which was 8 °C, did not affect blood biochemical parameters. It seems justified to use cyclo-ergometer tests due to the direct possibility of registering the mechanical parameters and reactions of soccer players in simulated climatic conditions, which cannot be tested on the pitch.

### Practical application

The results of this study can be applied in the design of training programmes to increase the physiological adaptations of players by simulating soccer-specific playing conditions for anaerobic capacity, specifically repeated maximal efforts. It is recommended that different protocols of repeated maximal efforts under high-temperature conditions be performed to increase tolerance to this type of effort. It is recommended that the national team arrive in Qatar as soon as possible after the end of league play. It is also advised to conduct a recovery camp during which the players will have earlier adapted to the climate, followed by a phase of direct preparation for the first match of the World Cup. This will allow for a later onset of the subjective feeling of fatigue, less change in the body’s acid–base balance, and allow for a minimum of power loss in the final phase of the game. In particular, these findings will be important during the upcoming 2022 World Cup in Qatar and in locations where high ambient temperatures are customary.

## Supplemental Information

10.7717/peerj.12658/supp-1 Supplemental Information 1 Database of mechanical parameters The following mechanical parameters were recorded in the study: Pmax, tmax, TW, Fi and RPE

10.7717/peerj.12658/supp-2 Supplemental Information 2 Database of biochemical parameters The parameters of the acid–base balance were determined, such as: pH, pCO2, pO2, HCO3, La-.

## Additional Information and Declarations

### Data Availability

The following information was supplied regarding data availability: The raw measurements are available in the Supplementary Files.

## References

[ref-1] Amonette W, Brown D, Dupler T, Xu J, Tufano J, De Witt J (2014). Physical determinants of interval sprint times in youth soccer players. Journal of Human Kinetics.

[ref-2] Andrade V, Santiago P, Kalva C, Campos EM, Papoti M (2016). Reproducibility of running anaerobic sprint test (RAST) for soccer players. Journal of Sports Medicine and Physical Fitness.

[ref-3] Andrzejewski M, Chmura J, Pluta B (2015). Sprinting activities and distance covered by top level Europa league soccer players. International Journal of Sports Science & Coaching.

[ref-4] Andrzejewski M, Chmura P, Konefał M, Kowalczuk E, Chmura J (2017). Match outcome and sprinting activities in match play by elite German soccer players. Journal of Sports Medicine and Physical Fitness.

[ref-5] Andrzejewski M, Konefał M, Chmura P, Kowalczuk E, Chmura J (2016). Match outcome and distances covered at various speeds in match play by elite German soccer players. International Journal of Performance Analysis in Sport.

[ref-6] Bangsbo J, Iaia F, Krustrup P (2007). Metabolic response and fatigue in soccer. International Journal of Sports Physiology and Performance.

[ref-7] Barnes C, Archer D, Hogg B, Bush M, Bradley P (2014). The evolution of physical and technical performance paramters in the english premier league. International Journal of Sports Medicine.

[ref-8] Billaut F, Smith K (2010). Prolonged repeated-sprint ability is related to arterial O_2_ desaturation in men. International Journal of Sports Physiology and Performance.

[ref-9] Chmura P, Andrzejewski M, Konefał M, Mroczek D, Rokita A, Chmura J (2017a). Analysis of motor activities of professional soccer players during the 2014 world cup in Brazil. Journal of Human Kinetics.

[ref-10] Chmura P, Konefał M, Andrzejewski M, Kosowski J, Rokita A, Chmura J (2017b). Physical activity profile of 2014 FIFA World Cup players, with regard to different ranges of air temperature and relative humidity. International Journal of Biometeorology.

[ref-11] Dolci F, Hart N, Kilding A, Chivers P, Piggott B, Spiteri T (2020). Physical and energetic demand of soccer: a brief review. Strength & Conditioning Journal.

[ref-12] Drust B, Rasmussen P, Mohr M, Nielsen B, Nybo L (2005). Elevations in core and muscle temperature impairs repeated sprint performance. Acta Physiologica Scandinavica.

[ref-13] Edge J, Bishop D, Hill-Haas S, Dawson B, Goodman C (2006). Comparison of muscle buffer capacity and repeated-sprint ability of untrained, endurance-trained and team-sport athletes. European Journal of Applied Physiology and Occupational Physiology.

[ref-14] Faude O, Koch T, Meyer T (2012). Straight sprinting is the most frequent action in goal situations in professional football. Journal of Sports Sciences.

[ref-15] Girard O, Bishop D, Racinais S (2013). Hot conditions improve power output during repeated cycling sprints without modifying neuromuscular fatigue characteristics. European Journal of Applied Physiology and Occupational Physiology.

[ref-16] Górski J (2006). Fizjologiczne podstawy wysiłku fizycznego.

[ref-17] Grantham J, Cheung S, Connes P, Febbraio M, Gaoua N, Gonza’lez Alonso J, Hue O, Johnson J, Maughan R, Meeusen R, Nybo L, Racinais S, Shirreffs S, Dvorak J (2010). Current knowledge on playing football in hot environments. Scandinavian Journal of Medicine & Science in Sports.

[ref-18] Kakamu T, Wada K, Smith D, Endo S, Fukushima T (2017). Preventing heat illness in the anticipated hot climate of the Tokyo 2020 Summer Olympic Games. Environmental Health and Preventive Medicine.

[ref-19] Kappenstein J, Engel F, Fernández-Fernández J, Ferrauti A (2015). Effects of active and passive recovery on blood lactate and blood pH after a repeated sprint protocol in children and adults. Pediatric Exercise Science.

[ref-20] Konefał M, Chmura P, Kowalczuk E, Figueiredo A, Sarmento H, Rokita A, Chmura J, Andrzejewski M (2019a). Modeling of relationships between physical and technical activities and match outcome in elite German soccer players. Journal of Sports Medicine and Physical Fitness.

[ref-21] Konefał M, Chmura P, Zając T, Chmura J, Kowalczuk E, Andrzejewski M (2019b). Evolution of technical activity in various playing positions, in relation to match outcomes in professional soccer. Biology of Sport.

[ref-22] Krustrup P, Mohr M, Steensberg A, Bencke J, Kjær M, Bangsbo J (2006). Muscle and blood metabolites during a soccer game: implications for sprint performance. Medicine and Science in Sports and Exercise.

[ref-23] Laursen P, Buchheit M (2019). Science and application of high-intensity interval training: solutions to the programming puzzle.

[ref-24] Linnane D, Bracken C, Brooks C, Cox V, Ball D (2004). Effects of hyperthermia on the metabolic responses to repeated high-intensity exercise. European Journal of Applied Physiology and Occupational Physiology.

[ref-25] Lucena R, Steinke E, Pacheco C, Vieira L, Betancour M, Steinke V (2017). The Brazilian World Cup: too hot for soccer?. International Journal of Biometeorology.

[ref-26] Maxwell B, Withers R, Ilsley A, Wakim M, Woods G, Day L (1998). Dynamic calibration of mechanically, air- and electromagnetically braked cycle ergometers. European Journal of Applied Physiology and Occupational Physiology.

[ref-27] McIntyre MC (2005). A comparison of the physiological profiles of elite Gaelic footballers, hurlers, and soccer players. British Journal of Sports Medicine.

[ref-28] Mendez-Villanueva A, Edge J, Suriano R, Hamer P, Bishop D (2012). The recovery of repeated-sprint exercise is associated with PCr resynthesis, while muscle pH and EMG amplitude remain depressed. PLOS ONE.

[ref-29] Mendez-Villanueva A, Hamer P, Bishop D (2008). Fatigue in repeated-sprint exercise is related to muscle power factors and reduced neuromuscular activity. European Journal of Applied Physiology and Occupational Physiology.

[ref-30] Mohr M, Krustrup P, Bangsbo J (2003). Match performance of high-standard soccer players with special reference to development of fatigue. Journal of Sports Sciences.

[ref-31] Mohr M, Mujika I, Santisteban J, Randers M, Bischoff R, Solano R, Hewitt A, Zubillaga A, Peltola E, Krustrup P (2010). Examination of fatigue development in elite soccer in a hot environment: a multi experimental approach. Scandinavian Journal of Medicine & Science in Sports.

[ref-32] Mohr M, Nybo L, Grantham J, Racinais S (2012). Physiological responses and physical performance during football in the heat. PLOS ONE.

[ref-33] Mohr M, Rasmussen P, Drust B, Nielsen B, Nybo L (2006). Environmental heat stress, hyperammonemia and nucleotide metabolism during intermittent exercise. European Journal of Applied Physiology and Occupational Physiology.

[ref-34] Nassis G, Brito J, Dvorak J, Chalabi H, Racinais S (2015). The association of environmental heat stress with performance: analysis of the 2014 FIFA World Cup Brazil. British Journal of Sports Medicine.

[ref-35] Ozgünen K, Kurdak S, Maughan R, Zeren C, Korkmaz S, Yazici Z, Ersöz G, Shirreffs S, Binnet M, Dvorak J (2010). Effect of hot environmental conditions on physical activity patterns and temperature response of football players. Scandinavian Journal of Medicine & Science in Sports.

[ref-36] Périard J, Pyne D, Bishop D, Wallett A, Girard O (2020). Short-term repeated-sprint training in hot and cool conditions similarly benefits performance in team-sport athletes. Frontiers in Physiology.

[ref-37] Racinais S, Bishop D, Denis R, Lattier G, Mendez-Villaneuva A, Perrey S (2007). Muscle deoxygenation and neural drive to the muscle during repeated sprint cycling. Medicine and Science in Sports and Exercise.

[ref-38] Racinais S, Blonc S, Jonville S, Hue O (2005). Time-of-day influences the environmental effects on muscle force and contractility. Medicine and Science in Sports and Exercise.

[ref-39] Racinais S, Cocking S, Périard J (2017). Sports and environmental temperature: from warming-up to heating-up. Temperature (Austin).

[ref-40] Racinais S, Hue O, Blonc S (2004). Time-of-day effects on anaerobic muscular power in a moderately warm environment. Chronobiology International.

[ref-41] Racinais S, Oksa J (2010). Temperature and neuromuscular function. Scandinavian Journal of Medicine & Science in Sports.

[ref-42] Racinais S, Wilson M, Périard J (2017). Passive heat acclimation improves skeletal muscle contractility in humans. American Journal of Physiology-Regulatory, Integrative and Comparative Physiology.

[ref-43] Robergs R, Ghiasv F, Parker D (2004). Biochemistry of exercise induced metabolic acidosis. American Journal of Physiology-Regulatory, Integrative and Comparative Physiology.

[ref-44] Vanos J, Thomas W, Grundstein A, Hosokawa Y, Liu Y, Casa D (2020). A multi-scalar climatological analysis in preparation for extreme heat at the Tokyo 2020 Olympic and Paralympic Games 2020. Termperature.

[ref-45] Yamaguchi K, Kasai N, Hayashi N, Yatsutani H, Girard O, Goto K (2020). Acute performance and physiological responses to repeated-sprint exercise in a combined hot and hypoxic environment. Physiological Reports.

